# Purification and characterisation of a quorum quenching AHL-lactonase from the endophytic bacterium *Enterobacter* sp. CS66

**DOI:** 10.1093/femsle/fny054

**Published:** 2018-03-06

**Authors:** Rajesh Padumane Shastry, Stephen K Dolan, Yassmin Abdelhamid, Ravishankar Rai Vittal, Martin Welch

**Affiliations:** 1 Department of Studies in Microbiology, University of Mysore, Manasagangotri, Mysore 570006, India; 2 Department of Biochemistry, University of Cambridge, Cambridge CB2 1QW, UK

**Keywords:** quorum sensing, *Pectobacterium atrosepticum*, quorum quenching

## Abstract

The quorum quenching (QQ) activity of endophytic bacteria associated with medicinal plants was explored. Extracts of the Gram-negative *Enterobacter* sp. CS66 possessed potent *N*-acylhomoserine lactone (AHL) hydrolytic activity *in vitro*. Using degenerate primers, we PCR-amplified an open reading frame (denoted *aiiE*) from CS66 that was 96% identical to the well-characterised AHL-lactonase AiiA from *Bacillus thuringiensis*, but only 30% was identical to AHL-lactonases from other Gram-negative species. This confirms that close AiiA homologs can be found in both Gram-positive and Gram-negative bacteria. Purified AiiE exhibited potent AHL-lactonase activity against a broad range of AHLs. Furthermore*, aiiE* was able to reduce the production of secreted plant cell wall-degrading hydrolytic enzymes when expressed *in trans* in the economically important plant pathogen, *Pectobacterium atrosepticum*. Our results indicate the presence of a novel AHL-lactonase in *Enterobacter* sp. CS66 with significant potential as a biocontrol agent.

## Teaser

A quorum quenching enzyme originally associated with Gram-positive bacterial species is also found in Gram-negative bacteria too.

**ABBREVIATIONS**
QS: quorum sensing
QQ: quorum quenching
AHL: *N*-acylated homoserine lactone
HEPES: 4-(2-hydroxyethyl)-1-piperazineethanesulfonic acid
Pel: pectate lyase

## INTRODUCTION

Many pathogens cause tissue damage by secreting a welter of exceptionally active proteases and phospholipases, and in many species of bacteria, the secretion of these exoproducts is now known to be coordinated by a cell–cell communication mechanism called ‘quorum sensing’ (QS). In essence, each cell in the population produces a set of inter-cellular signalling molecules (of which the best understood are the *N*-acylhomoserine lactones or AHLs), most of which are capable of freely diffusing into and out of cells (reviewed by Wang *et al.*^2006^). As the population cell density rises, so too does the bulk concentration of AHLs. This continues until a critical threshold concentration is achieved (related to the cell partitioning characteristics of the signal molecules and their affinity for cognate receptors). Unlike the situation in many other biological signalling systems, the AHL QS receptors are intracellular, and are almost universally comprised of a ligand-binding domain (LBD) coupled to a DNA-binding domain (Tsai and Winans 2010). Binding of the AHL to the LBD induces a conformational change that increases the affinity of the receptor protein for specific recognition sequences in the DNA (Welch *et al.*^2000^; Ventre *et al.*^2003^; Bottomley *et al.*^2007^). The activated receptor-DNA complex then stimulates (or, in some cases, represses) the transcription of the adjacent gene(s). In the case of pathogens, these genes often encode secreted exoenzymes and toxins associated with infection (Deep, Chaudhary and Gupta 2011). AHLs are synthesised by LuxI-type synthases, whereas the AHL receptors belong to the LuxR family of transcriptional regulators (LaSarre and Federle 2013). Although much attention has been paid to the role played by QS in controlling virulence in mammalian pathogens, it also plays a key role in controlling virulence in the plant pathogens too (reviewed by Von Bodman, Bauer and Coplin 2003). A textbook example of an AHL-based QS system has been demonstrated for the soft rotting plant pathogen *Pectobacterium atrosepticum* (Whitehead *et al.*^2002^).

*Pectobacterium atrosepticum* is a pathogen of potato, causing soft rot and blackleg disease (Baz *et al.*^2011^). In *P. atrosepticum*, QS controls the synthesis and secretion of a range of plant cell wall-degrading exoenzymes (PCWDEs), including proteases (Prt), cellulases (Cel) and pectate lyases (Pel). In addition, QS also impacts on O-antigen production (Bowden *et al.*2013b) and controls the expression and secretion of a harpin (HrpN), necrosis-inducing protein (Nip), and the antibiotic carbapenem, as well as a number of transcriptional regulators (ExpR, RsmA and VirR) (Liu *et al.*^2008^). The QS signal produced by *P. atrosepticum* is the AHL *N*-(3-oxohexanoyl)-L-homoserine lactone (OHHL), which is synthesised by ExpI. In concert with a rise in (p)ppGpp levels as nutrients become scarce, OHHL accumulation leads to increased expression of virulence-associated transcripts (Burr *et al.*^2006^; Bowden *et al.*2013a).

Because of the key role it plays in controlling virulence and biofilm formation by pathogenic bacteria, QS has become a popular target for the development of anti-virulence strategies. Some of these approaches employ small molecules to block QS molecule synthesis or reception (Hodgkinson *et al.*^2012^). For example, inhibition of QS lowers the production of virulence factors and depresses biofilm formation in both Gram-negative and Gram-positive bacterial pathogens (Hentzer and Givskov 2003). An alternative approach exploits the fact that some bacteria produce enzymes capable of degrading AHL signalling molecules. Such quorum quenching (QQ) enzymes exhibit AHL-lactonase, AHL-acylase, or AHL-oxidoreductase activity (Fetzner 2015). Of these, the AHL-lactonases have been most extensively studied, with examples including AiiA from a *Bacillus* sp. (Dong *et al.*^2000^), AhlD from *Arthrobacter* sp. IBN110 (Park *et al.*^2003^), AidC from *Chryseobacterium* sp. StRB126 (Wang *et al.*^2012^), AttM from *Agrobacterium tumefaciens* (Carlier *et al.*^2003^), AiiM from *Microbacterium testaceum* (Wang *et al.*^2010^) and AhlS from *Solibacillus silvestris* (Morohoshi *et al.*^2012^).

The microflora associated with many plants of medicinal interest have been under-investigated, and this environment remains a rich and unexploited reservoir of microbes with biotechnological potential. Recently, we reported on an *Enterobacter* sp. (denoted VT66) isolated from *Ventilago madraspatana*, which encodes a ca. 30 kDa enzyme with AHL-degrading activity (Rajesh and Rai 2015). However, the gene encoding the AHL degrading enzyme was not cloned, and the protein was only partially characterised. In the present study, we identified a gene encoding an AHL-lactonase from a different endophyte, *Enterobacter* sp. CS66. This gene (denoted *aiiE*) was cloned for recombinant expression and further characterisation, and its potential application in controlling the expression of virulence factors by the QS phytopathogen, *P. atrosepticum*, was explored.

## MATERIALS AND METHODS

### Bacterial strains and plasmids

The strains used in the study are listed in Table 1. Rosetta DE3 (derived from *Escherichia coli* BL21) was grown in the presence of chloramphenicol (Cm, 34 μg/mL) at 37°C. *Escherichia coli* JM109 was grown at 37°C. *Pectobacterium atrosepticum* wild type (strain Eca1043) and the isogenic *P. atrosepticum* mutant Δ*expI* (SB1031) were grown at 30°C. Where required, ampicillin (Ap) was used at a final concentration of 50 μg/mL. *Serratia* sp. SP19 and *Chromobacterium violaceum* CV026 were used as biosensors to detect C_4_-HSL. These strains were grown at 30°C.

**Table 1.** Bacterial strains and plasmids used in this study.

Strain or plasmid	Description	Source or references
Strains		
Rosetta DE3	*E. coli* BL21 derivative, pRare2, Cm^r^	Tegel *et al.*^2010^
*Serratia* sp. SP19	*Serratia* sp. ATCC 39006 derivative	Poulter *et al.*^2010^
*Escherichia. coli* JM109	*E. coli* strain for cloning and expression	New England Biolabs
*Enterobacter* sp. CS66	Endophytic AHL-degrading strain	This study
*Pectobacterium atrosepticum* (Eca1043)	Wild type	Bowden *et al.*2013a
*P. atrosepticum* (Δe*xpI*, SB1031)	Quorum sensing mutant	Bowden *et al.*2013a
Plasmids		
pMAL-c2X	MBP fusion cloning vector; Ap^r^	New England Biolabs
pET19m	Expression vector to make His_6_-tagged fusion proteins; Ap^r^ (modified pET-19b, Novagen)	Dolan *et al.*^2017^
pMAL-c2X-*aiiE*	pMAL containing *aiiE* from *Enterobacter* sp. CS66; Ap^r^	This study
pET19m-*aiiE*	pET19m containing *aiiE* from *Enterobacter* sp. CS66; Ap^r^	This study

### Isolation and identification of *Enterobacter* sp. CS66

A sample of *Coscinium fenestratum* Gaertn was collected from forest of Western Ghats in Karnataka, India (13.08°N, 75.45°E). The plant was identified by consulting taxonomists and the herbarium of the plant was preserved in the Department of Studies in Microbiology (MGMB/001/2013-14), University of Mysore, Mysore, India. Endophytic bacteria were isolated as previously described and screened for their ability to degrade AHLs (Rajesh and Rai 2015). Isolates capable of degrading AHLs were classified following 16S rDNA sequence analysis (Araújo *et al.*^2002^).

### Cloning and expression of *aiiE* from *Enterobacter* sp. CS66

The ORF encoding the AHL-degrading gene *aiiE* from *Enterobacter* sp. CS66 was cloned using a previously described method (Rajesh and Rai 2015). Briefly, the *aiiE* ORF was amplified from extracted genomic DNA using the polymerase chain reaction (PCR) with the forward primer 5^΄^- AAAGGATCCATGACAGTAAAGAAGCTTTATTTCAT-3^΄^ and the reverse primer 5^΄^- AAAGTCGACCTATATATACTCAGGGAACACTTTAC-3^΄^. These primers contained BamHI and SalI restriction sites (underlined) as indicated. The amplicon was digested with BamHI and SalI, and gel-purified. The digested fragment containing the *aiiE* ORF was then ligated to appropriately digested pMAL-c2X to yield pMAL-c2X-*aiiE*. Cultures of *E. coli* JM109 containing pMAL-c2X-*aiiE* were grown in LB medium at 37°C with good aeration (shaking at 200 rpm) until OD_600_ 0.5. The temperature was then lowered to 20°C, and isopropyl-β-D-thiogalactopyranoside (IPTG) was added to 1 mM final concentration to induce expression of the cloned gene. The induced culture was grown for a further 16 h before assaying the cells for AHL degradation activity.

### Purification of AiiE

The *aiiE*-coding region was PCR-amplified using the forward primer 5^΄^-AAACTCGAGATGACAGTAAAGAAGCTTTATTTCAT-3^΄^ and the reverse primer 5^΄^- AAAGGATCCCTATATATACTCAGGGAACACTTTAC-3^΄^. These primers contained XhoI and BamHI restriction sites (underlined) as indicated. Following digestion with XhoI and BamHI, the gel-purified amplicon was ligated to pET-19m that had been previously digested with the same enzymes and gel-purified. This yielded construct pET-19m-*aiiE*. For purification of the His_6_-tagged AiiE, the cells were grown in 1 L LB medium at 37°C with good aeration (shaking at 200 rpm) until OD_600_ 0.5. The temperature was then lowered to 20°C and IPTG was added to 0.5 mM final concentration to induce expression of the cloned gene. The induced culture was grown for a further 16 h and then harvested by centrifugation (6000× *g*, 4°C, 15 min). The cell pellet was resuspended in 20 mL of lysis buffer (50 mM sodium phosphate, 200 mM NaCl, 10% (v/v) glycerol, pH 8.0), and the cells were ruptured by sonication (3 × 10 s, Soniprep 150, maximum power output). The cell lysate was clarified by centrifugation (11 000× *g*, 4°C, 30 min), and the supernatant was filtered through a 0.45-μm filter. The filtered lysate was then loaded onto an Ni-NTA column (2 mL packed resin bed volume) and the column was washed overnight at 4°C with lysis buffer containing 10 mM imidazole. The His_6_-AiiE was eluted with lysis buffer containing 250 mM imidazole. The purified protein was dialyzed overnight against 2 L dialysis buffer (20 mM Tris-HCl, 50 mM NaCl, 5% (v/v) glycerol, 1 mM EDTA, 1 mM DTT, pH 7.5) in the presence of His_6_-tagged TEV-protease. The AiiE thus released was cleaned up by batch extraction in a slurry of Ni-NTA resin equilibrated in dialysis buffer. The purity of the AiiE was confirmed by SDS-PAGE, and loss of the His_6_-tag was confirmed by western blot analysis using commercially available anti-His_6_ antibodies.

### AHL lactonase activity

Two approaches were used to monitor the AHL degradation activity of AiiE. In the first, AHL levels were monitored using an agar overlay assay, as previously described (McClean *et al.*^1997^). Briefly, a 200 μL reaction containing 100 μM C_4_-HSL and 100 μg/mL purified AiiE or MBP-tagged AiiE was prepared in 10 mM potassium phosphate buffer (pH 7.2). The samples were incubated at 30°C for 6 h. Following this, the reaction mixture was heat inactivated (95°C for 10 min) and then filtered through a 0.45-μm filter. LB plates were overlaid with soft agar seeded with an overnight culture of either CV026 or *Serratia* SP19 (as indicated) and 5 μL of the heat-inactivated sterile reaction mixture was spotted onto the plates. The plates were then incubated at 30°C for 24 h to allow development of the pigment (violacein or prodigiosin, depending on the indicator strain used) halo.

For kinetic analyses, the catalytic activity of AiiE was measured spectrophotometrically as previously described (Liu *et al.*^2013^) with some modifications. Proton release from the hydrolysis of the AHL substrate was measured in weakly buffered solutions using the pH sensitive dye, phenol red. The 1 mL reaction mixture contained 1 mM HEPES, 200 mM NaCl, 50 μM phenol red (pH 7.5) and 0 to 10 mM C_4_-HSL substrate. The reaction was initiated by adding 10 μg AiiE. AHL hydrolysis was measured by monitoring the decrease in A_557_ over time. A standard curve was generated by titrating hydrochloric acid.

### Exoenzyme production by *P. atrosepticum*

The well-characterised wild-type strain, Eca1043, was used to examine whether AiiE affects secreted virulence factor production in *P. atrosepticum*. An isogenic AHL-deficient e*xpI* mutant, SB1031, served as a control. Plasmid pMAL-c2X harbouring the *aiiE* gene from *Enterobacter* sp. CS66 was introduced into each genetic background. Plasmid pMAL-c2X without the *aiiE* gene served as a control. Production of secreted pectate lyase (Pel) was monitored as previously described (Bowden *et al.*2013a). Overnight cultures of the *P. atrosepticum* strains were grown in LB supplemented with ampicillin (to maintain the plasmid) and 5 μL aliquots were spotted onto each Pel plates. The plates were then incubated at 30°C for 48 h. After incubation, plates were developed by flooding with 7.5% copper acetate to reveal the Pel halos. The production of secreted proteases (Prt) was followed using gelatin-agar plates, as previously described (Bowden *et al.*2013a). The Prt plates were inoculated as described for the Pel plates and incubated at 30°C for 48 h. The plates were developed by flooding with 4 M ammonium sulphate solution to reveal the halos.

### Statistical analyses

Virulence assays were analysed by one-way ANOVA using Graphpad Prism 5.03 software.

## RESULTS

### Identification of *aiiE* in *Enterobacter* sp. CS66

**Figure 1.**
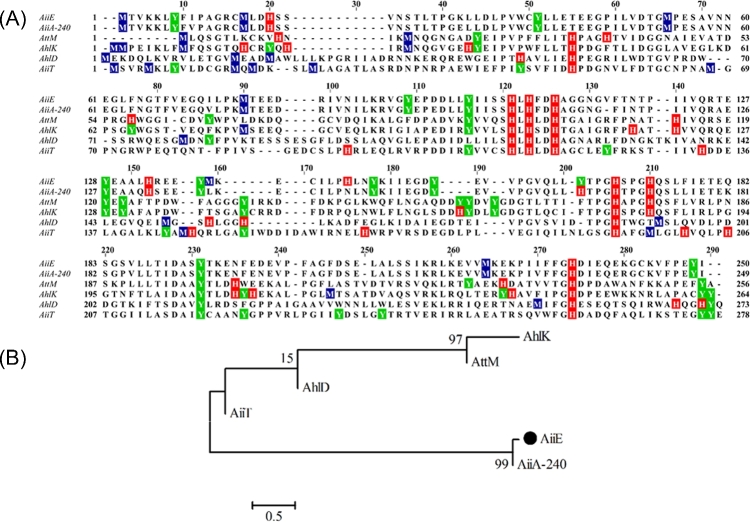
Sequence lineup showing the relationship between AiiA and other AHL-lactonases. (**A**) Multiple sequence alignment comparing AiiE from *Enterobacter* sp. CS66 with AiiA from *Bacillus* sp. 240B1 (AiiA-240, accession number: AF196486.1), AttM from *A. tumefaciens* C58 (AttM, accession number: Q7D3U0.3), AiiT from *Thermaerobacter composti* (AiiT, accession number: AB935248.1), AhlK from *Klebsiella pneumoniae* (AhlK, accession number: AY222324.1) and AhlD from *Arthrobacter* sp. IBN110 (AhlD, accession number: AF525800.1). Clustal Omega was used for alignment of sequences and Jalview was used to shade conserved histidine (red) and tyrosine (green) residues. The short conserved region H_104_XHXDH_109_ (AiiE numbering) represents the core dinuclear zinc binding motif. (**B**) Phylogenetic tree showing the evolutionary relationship between AiiE and the other AHL lactonases.

**Figure 2.**
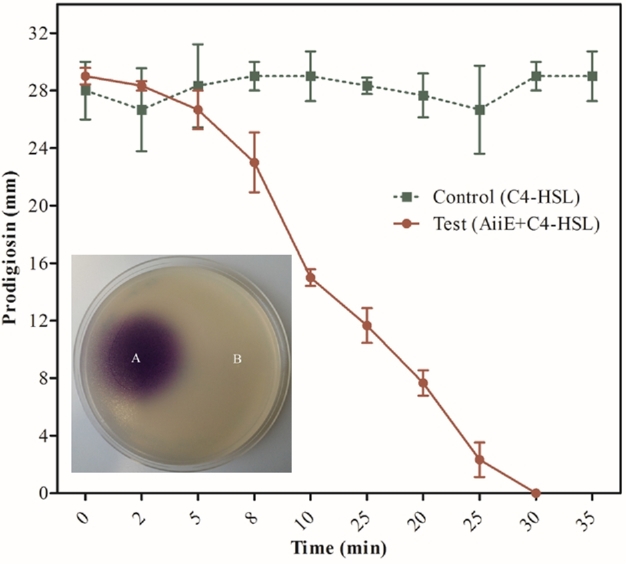
Purified AiiE rapidly degrades C_4_-HSL *in vitro*. The figure shows that 100 μg/mL (3.4 μM) AiiE can completely degrade 100 μM C_4_-HSL in just 30 min. C_4_-HSL levels were measured by monitoring the size of the prodigiosin halo obtained after spotting aliquots of the reaction mixture harvested at different times onto *Serratia* SP19 biosensor plates. Following development of the plates for 24 h, the diameter of the prodigios in halo (in mm) was measured. The control is C_4_-HSL incubated under otherwise identical conditions but in the absence of AiiE. Error bars represent the standard deviation of triplicate independent measurements. The inset shows a lawn of the C_4_-HSL biosensor strain, *C. violaceum* CV026, spotted with 5 μL of solution containing (**A**) 100 μM C_4_-HSL, or 5 μL solution containing (**B**) 100 μM C_4_-HSL treated with 100 μg/mL MBP-AiiE for 6 h.

An isolate of *Enterobacter* sp. CS66 was obtained from samples of the critically endangered tree, *Coscinium fenestratum* (also known as yellow vine or tree turmeric), a producer of the benzyliosoquinoline alkaloid, berberine. One of the endophytic bacteria (denoted *Enterobacter* sp. CS66) associated with the *C. fenestratum* samples was able to degrade AHLs (data not shown). Primers designed to anneal to the previously characterised *aiiA* gene from *Bacillus* sp. 240B1 (Dong *et al.*^2000^) were used to PCR-amplify a ca. 750 bp product from the genomic DNA of *Enterobacter* sp. CS66. BLAST analysis of the amplicon sequence revealed that the encoded ORF exhibited 92% identity at the amino acid level with the AiiA protein from *Bacillus* sp. 240B1, and 96% identity with the AiiA protein from *Bacillus thuringiensis* serovar *kurstaki*, which has been structurally characterised (Kim *et al.*^2005^). This was unexpected because close relatives of AiiA have not been reported in Gram-negative bacteria such as *Enterobacter* sp., and of the AHL-lactonases that have been identified in Gram-negative organisms; these share only distant similarity with AiiA. We therefore named the new gene *aiiE* (autoinducer inactivation gene from *Enterobacter*). A sequence alignment of AiiE against the best-characterised AHL-lactonases from a variety of organisms is shown in Fig. 1A, and a relationship tree is shown in Fig. 1B. The *aiiE* ORF was cloned into pMAL-c2X to generate an MBP-fusion protein, which was expressed and purified using an amylose column. The purified MBP-AiiE protein was able to completely degrade 100 μM C_4_-HSL within 6 h (Fig. 2, inset). This confirmed that AiiE is an AHL-lactonase with C_4_-HSL degradation activity.

### Expression and purification of AiiE

**Figure 3.**
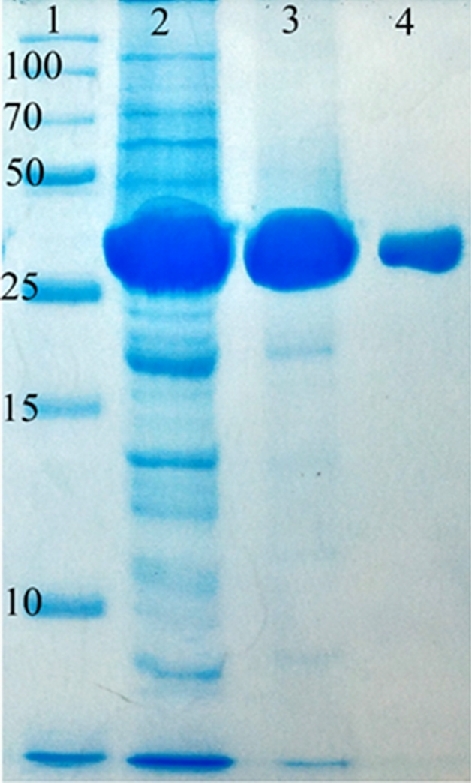
Purification of AiiE. The figure shows a Coomassie Brilliant Blue G250-stained 10% polyacrylamide gel run in SDS buffer showing the purification of AiiE. Lane 1; protein molecular marker, Lane 2; crude cell-free lysate, Lane 3; His_6_-tagged AiiE eluted from the Ni-NTA column, Lane 4; purified AiiE after His_6_-TEV protease cleavage.

His_6_-tagged AiiE was overexpressed from pET-19m in Rosetta DE3 cells and purified to homogeneity using an Ni-NTA affinity column. The His_6_-tag was removed using His_6_-tagged TEV protease. The purified protein was approximately 29 kDa in mass (Fig. 3), which was in agreement with the molecular mass of AiiE based on its predicted amino acid sequence. Western analysis (data not shown) confirmed that the purified protein no longer contained a His_6_-tag. To further characterise the purified protein, it was mixed with 100 μM C_4_-HSL incubated at 30°C. At different times, aliquots of the reaction mixture were withdrawn and assayed for their ability to restore production of red prodigiosin pigment by *Serratia* SP19. This strain is unable to produce prodiogiosin in the absence of exogenous AHL because it contains a mutation in the AHL synthase gene, *smaI*. However, in the presence of exogenous AHL (especially short chain AHLs such as C_4_-HSL), the amount of prodigiosin pigment produced is proportional to the concentration of AHL present. The sensitivity and dynamic range of SP19 is enhanced by the presence of additional mutations in the *pigX* and *pigZ* genes of the strain (Poulter *et al.*^2010^). AiiE was able to completely degrade a 10-fold molar excess of C_4_-HSL within 30 min (Fig. 2).

### Enzyme kinetics

To more accurately determine the steady state kinetic constants of AiiE, we measured the initial rates in the presence of AHL substrates with increasing acyl chain lengths (C_4_-HSL, C_6_-HSL, C_8_-HSL, C_10_-HSL and C_12_-HSL). Lineweaver-Burk plots (1/v_0_ versus 1/[S]) were used to determine the kinetic constants (k_cat_, K_m_) and the results are shown in Table 2. The k_cat_ values for all substrates were in the range of ca. 61 to 101 s^−1^, whereas the K_m_ values varied between ca. 6 and 15 mM. These values are very comparable with those reported previously for AiiA from *Bacillus* sp. B240 (Wang *et al.*^2004^). From the pseudo-second order rate constant (k_cat_/K_m_), it is clear that AiiE exhibits a slight preference for shorter chain AHLs. This contrasts with the findings of Wang *et al.* who reported that AiiA from *Bacillus* sp. B240 exhibits a slight preference for longer chain AHLs, although in both studies, the acyl chain length preference is only marginal (Wang *et al.*^2004^).

**Table 2.** Steady-state kinetic constants of AHL hydrolysis.

AHL-lactonase	Substrate	*k*_cat_ (s^−1^)	*K_m_* (mM)	*k*_cat_/*K_m_* (mM^−1^ s^−1)^
AiiE	C_4_-HSL	61.14	6.01	10.17
	C_6_-HSL	69.43	7.88	8.81
	C_8_-HSL	80.07	10.35	7.74
	C_10_-HSL	98.31	14.88	6.61
	C_12_-HSL	101.5	15.21	6.67

### AiiE expression very effectively depresses virulence factor expression in *P. atrosepticum*

**Figure 4.**
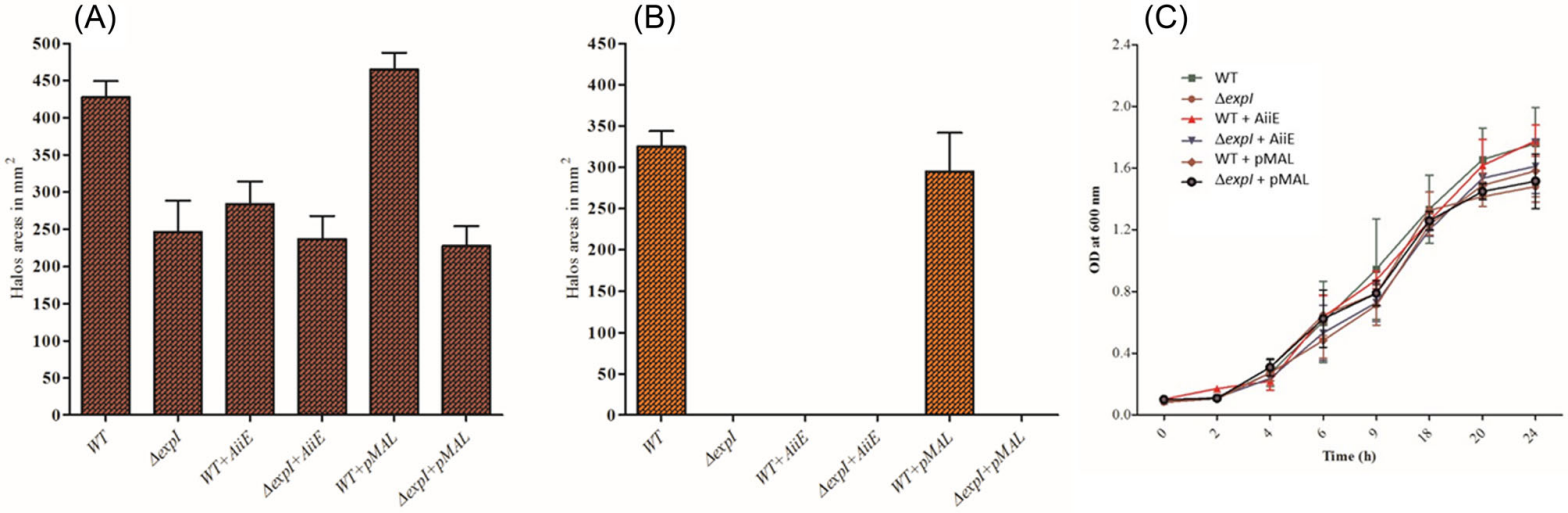
Inhibition of virulence factor expression in *P. atrosepticum* by AiiE. (**A**) Secreted pectate lyase (Pel) activity; (**B**) secreted protease (Prt) activity; (**C**) growth curves of the indicated strains. The Pel and Prt activities are expressed as the area of the halo (in mm^2^) around the point of bacterial inoculation on Pel and Prt plates, respectively. WT, *P. atrosepticum* strain Eca1043; Δ*expI*, *P. atrosepticum expI* mutant (SB1031); WT + AiiE, *P. atrosepticum* strain Eca1043 containing pMAL-c2X-*aiiE*; Δ*expI *+ AiiE, SB1031 containing pMAL-c2X-*aiiE*; WT + pMAL, *P. atrosepticum* strain Eca1043 containing pMAL-c2X; Δ*expI* + pMAL, SB1031 containing pMAL-c2X.

When AiiE was expressed from a plasmid *in trans* in wild-type *P. atrosepticum*, the production of secreted pectate lyase (Pel) and secreted protease (Prt) diminished to levels equivalent to that of an OHHL-deficient *expI* mutant (Fig. 4A and B). In contrast, Pel and Prt production by the wild-type strain containing an empty vector (pMAL-c2X) was unaffected. As a further control, we also examined whether the presence of the plasmid (pMAL-c2X) or AiiA had any effect on growth; it did not (Fig. 4C). We conclude that expression of *aiiE in trans* in *P. atrosepticum* abolishes production of secreted protease (Prt) and reduces production of secreted pectate lyase (Pel) to levels equivalent to that of an *expI* mutant.

## DISCUSSION

An isolate of the Gram-negative endophytic *Enterobacter* sp. CS66 was identified. This isolate was capable of rapidly degrading exogenously supplied AHLs, and this activity was associated with an AiiA-like protein, denoted here as AiiE. The first AHL-lactonase to be described, AiiA, was originally isolated from a Gram-positive soil-dwelling bacterium, *Bacillus* sp. 240B1. Remarkably, sequence comparisons revealed that AiiA and AiiE are 90% identical at the amino acid level (rising to 96% when comparing AiiE with the more recently characterised AiiA enzyme from *B. thuringiensis* serovar *kurstaki*), suggesting that the gene encoding these enzymes may have been acquired by relatively recent horizontal gene transfer, either between species or from a common source. Although this is not the first discovery of an AiiA homolog in Gram-negative bacteria—the AttM protein from *A. tumefaciens* is also an AHL lactonase (Zhang, Wang and Zhang 2002)—it is worth noting that AttM and AiiA are only distantly related (sharing just 31% amino acid identity). Furthermore, other AHL-lactonases from Gram-negative bacteria, such as the AhlK protein from *Klebsiella pneumoniae*, are much more similar to AttM than they are to AiiA/AiiE (Fig. 1B). It is therefore surprising to find two almost identical AHL-lactonases conserved between Gram-positive and Gram-negative species.

AiiE showed broad specificity, although it exhibited a preference for hydrolysing shorter chain AHLs. Consistent with a previous study reporting on the kinetics of AiiA from *Bacillus* sp. B240 (Wang *et al.*^2004^), the very best k_cat_/K_m_ value measured here (6012 M^−1^ s^−1^, for C_4_-HSL) is much lower than the diffusion limit (10^8^–10^9^ M^−1^ s^−1^), suggesting that the enzyme is inefficient and that AHLs are unlikely to be its ‘true’ substrate. Nevertheless, AiiE contained all the conserved amino acids known to be required for AHL-lactonase activity (especially the dinuclear zinc binding motif ‘HXHXDH’ [Thomas *et al.*^2005^; Liao, Yu and Himo 2009]). Furthermore, it needs to be recognised that AHL degradation may not be the primary physiological function of these enzymes. Indeed, in the case of the AHL lactonase AttM (also known as BlcC), it seems likely that its main function is to convert γ-butyrolactone to γ-hydroxybutyrate (Chain *et al.*^2007^). We did test cultures of *Enterobacter* sp. CS66 to see whether they produce detectable AHLs, but none were identified. However, and as pointed out by Chan *et al.* (2011), in polymicrobial communities such as the soil or rhizosphere, AHL-lactonases enzymes are capable of degrading both self-produced and exogenous AHLs, so it remains formally possible that one function of AiiE is in scavenging carbon via AHL degradation from the community.

Despite its relatively low catalytic efficiency, the AHL-lactonase activity of AiiE was more than enough to effectively disrupt QS-dependent virulence factor production by *P. atrosepticum*. Expression of *aiiE* from pMAL-c2X completely abolished protease secretion (a phenotype which is under tight QS control) and decreased secreted pectate lyase production (a phenotype which is under partial QS control) to levels comparable with that of an isogenic *expI* mutant. This suggests that AiiE (or organisms expressing *aiiE*) has the potential to be used as a biocontrol agent to depress pathogenicity in those pectolytic phytopathogens that employ QS to regulate virulence. This notwithstanding, and as noted above though, the enzyme is not especially active as an AHL-lactonase, and may need additional engineering to improve its catalytic parameters. In this regard, we are currently working towards solving the structure of the AiiE enzyme, with a view towards using the structural data as a template to direct rational modification of the protein to improve catalysis and specificity.

## FUNDING

The authors are thankful to Department of Science and Technology, New Delhi, for providing a fellowship for RPS (Grant order number DST/INSPIRE Fellowship/2011(265), 17 July 2011) and for the award of Newton Bhabha PhD Placement Programme-2016 supporting RPS during his secondment to the laboratory of MW. YA is supported by the Yousef Jameel Foundation. SKD is supported by the BBSRC. Work on quorum sensing in the MW laboratory is supported by the BBSRC and the Rosetrees Trust.

***Conflict of interest***. None declared.

## References

[bib1] Araújo WL, Marcon J, Maccheroni W Diversity of endophytic bacterial populations and their interaction with xylella fastidiosa in citrus plants. Appl Environ Microb 2002;68:4906–14. 10.1128/AEM.68.10.4906-4914.2002 PMC126398 12324338

[bib2] Baz M, Lahbabi D, Samri S Control of potato soft rot caused by *Pectobacterium carotovorum* and *Pectobacterium atrosepticum* by Moroccan actinobacteria isolates. World J Microb Biot 2012;28:303–11. 10.1007/s11274-011-0820-5 22806806

[bib3] Bottomley MJ, Muraglia E, Bazzo R Molecular insights into quorum sensing in the human pathogen *Pseudomonas aeruginosa* from the structure of the virulence regulator LasR bound to its autoinducer. J Biol Chem 2007;282:13592–600. 17363368 10.1074/jbc.M700556200

[bib4] Bowden SD, Eyres A, Chung JCS Virulence in *Pectobacterium atrosepticum* is regulated by a coincidence circuit involving quorum sensing and the stress alarmone, (p)ppGpp. Mol Microbiol2013a;90:457–71. 23957692 10.1111/mmi.12369

[bib5] Bowden SD, Hale N, Chung JCS Surface swarming motility by *Pectobacterium atrosepticum* is a latent phenotype that requires O antigen and is regulated by quorum sensing. Microbiology2013b;159:2375–85. 24025601 10.1099/mic.0.070748-0

[bib6] Burr T, Barnard AML, Corbett MJ Identification of the central quorum sensing regulator of virulence in the enteric phytopathogen, *Erwinia carotovora*: the VirR repressor. Mol Microbiol 2006;59:113–25. 16359322 10.1111/j.1365-2958.2005.04939.x

[bib7] Carlier A, Uroz S, Smadja B The Ti plasmid of *Agrobacterium tumefaciens* harbors an attM-Paralogous Gene, aiiB, also encoding N-acyl homoserine lactonase activity. Appl Environ Microb 2003;69:4989–93. 10.1128/AEM.69.8.4989-4993.2003 PMC169067 12902298

[bib8] Chai Y, Tsai CS, Cho H Reconstitution of the biochemical activities of the AttJ repressor and the AttK, AttL, and AttM catabolic enzymes of *Agrobacterium tumefaciens*. J Bacteriol 2007;189:3674–9. 17307843 10.1128/JB.01274-06 PMC1855881

[bib9] Chan KG, Atkinson S, Mathee K Characterization of N-acylhomoserine lactone-degrading bacteria associated with the *Zingiber officinale* (ginger) rhizosphere: co-existence of quorum quenching and quorum sensing in Acinetobacter and Burkholderia. BMC Microbiol 2011;11:51. 21385437 10.1186/1471-2180-11-51 PMC3062576

[bib10] Deep A, Chaudhary U, Gupta V Quorum sensing and bacterial pathogenicity: from molecules to disease. J Lab Phys 2011;3:4–11. 10.4103/0974-2727.78553 PMC3118056 21701655

[bib11] Dolan SK, Bock T, Hering V Structural, mechanistic and functional insight into gliotoxin bis-thiomethylation in *Aspergillus fumigatus*. Open Biol 2017;7:pii:160292. 10.1098/rsob.160292 PMC5356443 28179499

[bib12] Dong Y-H, Xu J-L, Li X-Z AiiA, an enzyme that inactivates the acylhomoserine lactone quorum-sensing signal and attenuates the virulence of Erwinia carotovora. P Natl Acad Sci USA 2000;97:3526–31. 10.1073/pnas.060023897 PMC16273 10716724

[bib13] Fetzner S Quorum quenching enzymes. J Biotechnol 2015;201:2–14. 25220028 10.1016/j.jbiotec.2014.09.001

[bib14] Hentzer M, Givskov M Pharmacological inhibition of quorum sensing for the treatment of chronic bacterial infections. J Clin Invest 2003;112:1300–7. 14597754 10.1172/JCI20074 PMC228474

[bib15] Hodgkinson JT, Galloway WRJD, Wright M Design, synthesis and biological evaluation of non-natural modulators of quorum sensing in *Pseudomonas aeruginosa*. Org Biomol Chem 2012;10:6032–44. 22499353 10.1039/c2ob25198a

[bib16] Kim MH, Choi WC, Kang HO The molecular structure and catalytic mechanism of a quorum-quenching N-acyl-L-homoserine lactone hydrolase. P Natl Acad Sci USA 2005;102:17606–11. 10.1073/pnas.0504996102 PMC1295591 16314577

[bib17] LaSarre B, Federle MJ Exploiting quorum sensing to confuse bacterial pathogens. Microbiol Mol Biol Rev 2013;77:73–111. 23471618 10.1128/MMBR.00046-12 PMC3591984

[bib19] Liao R-Z, Yu J-G, Himo F Reaction mechanism of the dinuclear zinc enzyme N-Acyl-l-homoserine lactone Hydrolase: a quantum chemical study. Inorg Chem 2009;48:1442–8. 19159270 10.1021/ic801531n

[bib20] Liu CF, Liu D, Momb J A phenylalanine clamp controls substrate specificity in the quorum-quenching metallo-γ-lactonase from *Bacillus thuringiensis*. Biochemistry 2013;52:1603–10. 23387521 10.1021/bi400050j PMC3603367

[bib21] Liu H, Coulthurst SJ, Pritchard L Quorum sensing coordinates brute force and stealth modes of infection in the plant pathogen *Pectobacterium atrosepticum*. PLoS Pathog 2008;4:e1000093. 18566662 10.1371/journal.ppat.1000093 PMC2413422

[bib22] McClean KH, Winson MK, Fish L Quorum sensing and *Chromobacterium violaceum*: exploitation of violacein production and inhibition for the detection of N-acylhomoserine lactones. Microbiology 1997;143:3703–11. 9421896 10.1099/00221287-143-12-3703

[bib23] Morohoshi T, Tominaga Y, Someya N Complete genome sequence and characterization of the N-acylhomoserine lactone-degrading gene of the potato leaf-associated *Solibacillus silvestris*. J Biosci Bioeng 2012;113:20–5. 22019407 10.1016/j.jbiosc.2011.09.006

[bib24] Park S-Y, Lee SJ, Oh T-K AhlD, an N-acylhomoserine lactonase in Arthrobacter sp., and predicted homologues in other bacteria. Microbiology 2003;149:1541–50. 12777494 10.1099/mic.0.26269-0

[bib25] Poulter S, Carlton TM, Su X Engineering of new prodigiosin-based biosensors of Serratia for facile detection of short-chain N-acyl homoserine lactone quorum-sensing molecules. Environ Microbiol Rep 2010;2:322–8. 23766084 10.1111/j.1758-2229.2010.00140.x

[bib26] Rajesh PS, Rai VR Purification and antibiofilm activity of AHL-lactonase from endophytic *Enterobacter aerogenes* VT66. Int J Biol Macromol 2015;81:1046–52. 26432367 10.1016/j.ijbiomac.2015.09.048

[bib28] Tegel H, Tourle S, Ottosson J Increased levels of recombinant human proteins with the *Escherichia coli* strain Rosetta(DE3). Protein Expr Purif 2010;69:159–67. 19733669 10.1016/j.pep.2009.08.017

[bib29] Thomas PW, Stone EM, Costello AL The quorum-quenching lactonase from *Bacillus thuringiensis* is a metalloprotein. Biochemistry 2005;44:7559–69. 15895999 10.1021/bi050050m

[bib30] Tsai CS, Winans SC LuxR-type quorum-sensing regulators that are detached from common scents. Mol Microbiol 2010;77:1072–82. 20624221 10.1111/j.1365-2958.2010.07279.x PMC2975784

[bib27] Ventre I, Ledgham F, Prima V Dimerization of the quorum sensing regulator RhlR: development of a method using EGFP fluorescence anisotropy. Mol Microbiol 2003;48:187–98. 12657054 10.1046/j.1365-2958.2003.03422.x

[bib31] Von Bodman SB, Bauer WD, Coplin DL Quorum sensing in plant-pathogenic bacteria. Annu Rev Phytopathol 2003;41:455–82. 12730390 10.1146/annurev.phyto.41.052002.095652

[bib32] Wang JH, Smith D, Swatton JE Quorum sensing in Gram-negative bacteria. Sci Prog 2006;89:167–211. 17338438 10.3184/003685006783238335 PMC10368359

[bib33] Wang LH, Weng LX, Dong YH Specificity and enzyme kinetics of the quorum-quenching *N*-Acyl homoserine lactone lactonase (AHL-lactonase). J Biol Chem 2004;279:13645–51. 14734559 10.1074/jbc.M311194200

[bib34] Wang W-Z, Morohoshi T, Ikenoya M AiiM, a novel class of N-acylhomoserine lactonase from the leaf-associated bacterium *Microbacterium testaceum*. Appl Environ Microb 2010;76:2524–30. 10.1128/AEM.02738-09 PMC2849219 20173075

[bib35] Wang W-Z, Morohoshi T, Someya N AidC, a novel N-Acylhomoserine lactonase from the potato root-associated cytophaga-flavobacteria-bacteroides (CFB) group bacterium chryseobacterium sp. strain StRB126. Appl Environ Microb 2012;78:7985–92. 10.1128/AEM.02188-12 PMC3485932 22941089

[bib36] Welch M, Todd DE, Whitehead NA N-acyl homoserine lactone binding to the CarR receptor determines quorum-sensing specificity in Erwinia. EMBO J 2000;19:631–41. 10675332 10.1093/emboj/19.4.631 PMC305601

[bib37] Whitehead NA, Byers JT, Commander P The regulation of virulence in phytopathogenic *Erwinia* species: quorum sensing, antibiotics and ecological considerations. Ant Van Lee 2002;81:223–31. 10.1023/a:1020570802717 12448721

[bib38] Zhang HB, Wang LH, Zhang L-H Genetic control of quorum-sensing signal turnover in *Agrobacterium tumefaciens*. P Natl Acad Sci USA 2002;99:4638–43. 10.1073/pnas.022056699 PMC123700 11930013

